# Avian Influenza A(H7N9) Virus Infection in 2 Travelers Returning from China to Canada, January 2015^1^

**DOI:** 10.3201/eid2201.151330

**Published:** 2016-01

**Authors:** Danuta M. Skowronski, Catharine Chambers, Reka Gustafson, Dale B. Purych, Patrick Tang, Nathalie Bastien, Mel Krajden, Yan Li

**Affiliations:** British Columbia Centre for Disease Control, Vancouver, British Columbia, Canada (D.M. Skowronski, C. Chambers, P. Tang, M. Krajden);; University of British Columbia, Vancouver (D.M. Skowronski, R. Gustafson, D.B. Purych, P. Tang, M. Krajden);; Vancouver Coastal Health Authority, Vancouver (R. Gustafson);; LifeLabs Medical Laboratories, Surrey, British Columbia, Canada (D.B. Purych);; Public Health Agency of Canada, Winnipeg, Manitoba, Canada (N. Bastien, Y. Li);; University of Manitoba, Winnipeg (Y. Li)

**Keywords:** Influenza A Virus, subtype H7N9, influenza A(H7N9), avian influenza, influenza, humans, hemagglutination inhibition tests, microneutralization assay, Canada, travelers, viruses, serosurvey, epidemiology, virologic features

## Abstract

In January 2015, British Columbia, Canada, reported avian influenza A(H7N9) virus infection in 2 travelers returning from China who sought outpatient care for typical influenza-like illness. There was no further spread, but serosurvey findings showed broad population susceptibility to H7N9 virus. Travel history and timely notification are critical to emerging pathogen detection and response.

Since February 2013, >600 human cases of avian influenza A(H7N9) infection have been reported from eastern China, where the virus is considered enzootic in poultry (*1*). Travel-associated cases have previously been reported in Asia (*2*); however, in January 2015, Canada reported 2 travel-associated cases, which are described here.

## The Study

A married couple, both 56 years of age, from British Columbia (BC), Canada, traveled in Hong Kong (December 29, 2014–January 3, 2015, and January 6–7); Taipei, Taiwan (January 3–6); and Fujian Province, China (January 7–11), returning home on January 12. They recalled seeing live poultry and copious droppings while visiting Fujian on January 8 but recollected no other poultry contact.

Around January 3–7, the previously healthy woman experienced mild cough, sore throat, and hoarseness. She recovered, but influenza-like illness (ILI), including fever, cough, myalgia, and fatigue, developed on January 14. On January 15, she sought outpatient care. A healthcare worker (HCW) collected a nasal swab specimen, which was sent to the BC Public Health Microbiology and Reference Laboratory (BC-PHMRL), where most influenza testing is centralized in BC. On January 16, the sample tested positive for influenza A virus by reverse transcription PCR (RT-PCR), and the HCW was informed; the next business day (January 19), the patient was prescribed oseltamivir.

For surveillance purposes, BC-PHMRL conducts subtyping of all detected influenza A viruses. Despite a high virus titer (cycle threshold [C_t_] 23.43), the specimen could not be subtyped for human influenza H1 or H3 virus by matrix gene–based RT-PCR. Further subtyping using RT-PCR–based targets for the hemagglutinin gene indicated an H7 virus. Sequence analysis of a matrix gene fragment showed 99% identity with H7N9 and H9N2 viruses, the latter of which is known to have donated internal genes to H7N9 virus (*1**,**3*). On January 23, BC-PHMRL notified public health authorities of a presumptive diagnosis of H7N9 virus infection in the woman (index case), and on January 26, Canada’s National Microbiology Laboratory (NML) confirmed the diagnosis by RT-PCR.

On January 13, a day before the woman became ill, her husband, who had a history of asthma, had onset of ILI symptoms (fever, productive cough, chest pain, dyspnea, headache, myalgia, and fatigue) and visited the same HCW. The HCW prescribed doxycycline but did not collect a specimen. On January 19, after his wife received a diagnosis of influenza, the man was prescribed oseltamivir. A throat swab specimen collected from him on January 23 was RT-PCR–positive for influenza A (C_t_ 29.79). On January 29, NML confirmed H7N9 virus infection.

Neither patient experienced conjunctivitis, which has been reported with H7N7 and H7N3 infections (*3*,*4*), and both recovered in self-isolation at home. Follow-up respiratory specimens were still positive by RT-PCR on January 26 (day 12 after ILI onset for the woman [C_t_ 36.82]; day 13 for the man [C_t_ 33.80]) and on January 28 (day 15 after ILI onset) for the man (C_t_ 30.21).

The HCW remained asymptomatic; however, because of the patients’ travel history, the HCW began oseltamivir prophylaxis after learning the index patient had laboratory-confirmed influenza. Throat swab specimens collected from the HCW on January 26 were influenza RT-PCR–negative. Approximately 20 other close contacts of the patients were placed under 10-day surveillance (from last exposure), including 1 who received oseltamivir prophylaxis. All contacts remained asymptomatic. Passengers on the flight taken by the patients while they were asymptomatic were not included in active surveillance because >10 days had elapsed (*5*); however, media communications included flight details and public health advice.

Virus cultures in Madin-Darby canine kidney cells were attempted with all patient samples; only the woman’s January 15 sample was culture-positive for influenza virus (A/British Columbia/1/2015[H7N9]). Phylogenetic analysis of the hemagglutinin and neuraminidase genes showed that the strain clustered with 2014 and 2015 H7N9 human isolates from Jiangsu, Zhejiang, and Fujian Provinces, China, and 2014 chicken isolates from Jiangxi, China, belonging to clade W2-C (*1*). Similar to genomes of previous human H7N9 isolates, the genome of the patient’s isolate showed clinically relevant markers: substitutions G186V (H3 numbering), Q226L, and T160L in the hemagglutinin for increased human receptor affinity; substitution E627K in polymerase basic 2 for mammalian replication; substitutions S31N and V27I in matrix 2 for amantadine resistance; deletion in the neuraminidase stalk (positions 69–73); and neuraminidase inhibitor susceptibility (*3*).

Antibody titers to H7N9 and recent human influenza A(H3N2) and A(H1N1)pdm09 strains in paired serum samples from the case-patients and HCW were measured by hemagglutination inhibition assay at NML (Table 1) (*6*). At ≈7 weeks after ILI onset, the case-patients showed seroconversion (>4-fold antibody rise) to H7N9 virus; the HCW had no detectable H7N9 antibody (Table 1).

**Table 1:** Antibody titers to avian influenza A(H7N9) virus and recent human influenza A(H3N2) and A(H1N1)pdm09 virus strains for 2 persons with virologically confirmed H7N9 virus infection and for an HCW contact, British Columbia, Canada, January 2015*

Person, date of specimen collection	Duplicate inverse HI titers and GMT for influenza antibody										
	A/British Columbia/1/2015(H7N9)†				A/Switzerland/9715293/2013(H3N2)‡§				A/California/07/2009(H1N1)‡¶		
	Titer 1	Titer 2	GMT		Titer 1	Titer 2	GMT		Titer 1	Titer 2	GMT
Index patient#											
January 26	20	20	20		10	10	10		10	10	10
March 5	80	160	113		10	10	10		10	10	10
Second patient#											
January 26	20	40	28		10	10	10		10	10	10
March 5	160	160	160		20	20	20		20	20	20
HCW contact**											
January 27	<10	<10	<10		40	40	40		20	20	20
May 1	<10	<10	<10		40	40	40		20	20	20

*The 2 H7N9 virus–infected persons were a married couple; the woman was the index patient, and the man was the second patient. GMT, geometric mean titer; HCW, healthcare worker; HI, hemagglutination inhibition. †Assay was conducted by using homologous H7N9 virus isolated from the index patient (Global Initiative on Sharing Avian Influenza Data accession no. EPI_ISL_171342); the virus was antigenically equivalent to influenza A/Anhui/1/2013(H7N9), which was used in the population serosurvey reported in Table 2. The HI assay was conducted by using horse erythrocytes, as previously described (*6*). ‡Assay was conducted by using viruses of each human influenza A H1 and H3 subtype to which strains identified globally during the 2014–15 influenza season were considered antigenically related (see http://www.who.int/influenza/vaccines/virus/recommendations/2015_16_north/en/). Titers were measured according to standard assay protocols of the National Microbiology Laboratory, Canada’s influenza reference laboratory. §Assay was conducted by using guinea pig erythrocytes and in the presence of oseltamivir carboxylate to address potential neuraminidase-mediated binding of influenza A(H3N2) viruses to erythrocytes. ¶Assay was conducted by using turkey erythrocytes. #Received neither the 2013–14 nor 2014–15 influenza vaccine nor prior pneumococcal vaccine. **Received the 2013–14 and the 2014–15 influenza vaccines.

As part of population risk assessment, we also previously measured hemagglutination inhibition antibody titers to H7N9 virus in anonymized residual serum samples collected and banked from patients attending community-based laboratory test sites in 2010 (n = 1,116; ≈100 samples/10-year age group) (*7*) and 2013 (n = 496; ≈50 samples/10-year age group) (*8*) across the same BC region to which the couple returned. The assessment was conducted as described (*7**,**8*) and approved by the University of British Columbia Research Ethics Board. Results showed broad population serosusceptibility to H7N9 virus: 5% (10/201) of serum samples collected from patients >60 years of age in 2013 had low-level titers (>10 but <40), but no other samples had detectable H7N9 virus antibody (Table 2). Three samples with titers >20 showed titers <10 by microneutralization assay (Table 2).

**Table 2:** Antibody titers to influenza A/Anhui/1/2013(H7N9) in an anonymized population serosurvey, Lower Mainland, British Columbia, Canada, May 2013*

Age group, y	No. patients†	Median age, y	% Female	Mean GMT (95% CI)‡	No. (%, 95% CI)§	
					With titer >10	With titer >20
<10	49	4	47	5	0	0
10–19	48	16	67	5	0	0
20–29	49	27	69	5	0	0
30–39	50	34	90	5	0	0
40–49	50	46	60	5	0	0
50–59	49	55	49	5	0	0
60–69	50	65.5	56	5.3 (4.9–5.7)	3 (6.0, 0.0–12.6)	1 (2.0, 0–5.9)¶
70–79	50	75	42	5	0	0
80–89	50	83	40	5.4 (5–5.9)	4 (8.0, 0.5–15.5)	1 (2.0, 0–5.9)¶
>90	51	92	73	5.3 (4.9–5.6)	3 (5.9, 0.0–12.4)	1 (2.0, 0–5.8)¶
All	496	50	59	5.1 (5–5.2)	10 (0.9, 0.2–1.7)#	3 (0.3, −0.1 to 0.7)#

*Titers were measured by hemagglutination inhibition assay by using horse erythrocytes as previously described (*6*); assays were conducted at the National Microbiology Laboratory, Canada’s influenza reference laboratory in July 2013. GMT, geometric mean titer. †Serum samples were collected in May 2013; 5 samples had insufficient serum and were excluded from the analyses (*8*). ‡Titers were assessed in duplicate. Titers <10 were assigned a value of 5. GMT of duplicate titers derived as individual titers and group GMTs derived by age and overall. §No patients had a titer >40. ¶Further assessed by microneutralization (MN) assay, according to procedures described by the Centers for Disease Control and Prevention (Atlanta, GA, USA); available by request. MN titers for all 3 samples were <10. #Age-standardized (direct method) to the 2013 Fraser Valley and Greater Vancouver, British Columbia, Canada, population projections (BC Stats, 2013: http://www.bcstats.gov.bc.ca/StatisticsBySubject/Demography/PopulationProjections.aspx).

## Conclusions

The onset of ILI in 2 BC patients 5–6 days after observing poultry in China is consistent with the median 5-day incubation period reported elsewhere for H7N9 virus (*9*) and with common-source acquisition of the virus in Fujian. However, we cannot rule out other unrecognized exposures or person-to-person transmission between the couple. Continued RT-PCR detection in respiratory specimens 2 weeks after ILI onset has been documented (*9*) but does not necessarily signify ongoing shedding of infectious virus. Although previous case series have reported inconsistent antibody responses to H7N9 virus, often with low avidity and potentially correlated with clinical outcome (*10**–**12*), both BC case-patients with typical outpatient ILI demonstrated seroconversion.

Reported human cases of H7N9 infection have mostly been in older men, and two thirds have been categorized as severe (*2**,**3**,**9*). Mild illness has occasionally been reported in children (*2**,**13**,**14*), but, as exemplified by the BC cases, adults can also experience milder infection. Imported cases of novel influenza are less likely to be recognized if they are mild. In that regard, identification of H7N9 virus in an outpatient setting was adventitious. Travel history triggered specimen collection by the HCW, and identification of nonsubtypeable influenza by the provincial laboratory prompted further investigation and public health notification.

Despite broad susceptibility and instances of household or familial transmission, H7N9 virus has not demonstrated easy person-to-person spread. Poultry exposure remains the major risk factor for human H7N9 infection (*2**,**3**,**9*). Primary prevention messages should emphasize to travelers that they avoid exposure to poultry and uncooked poultry products while visiting affected areas. As illustrated by a prior imported case of avian influenza A(H5N1) virus to Alberta, Canada, however, such exposures may not always be recognized or avoidable (*15*). Screening should therefore begin with travel history in the 2 weeks before onset of acute respiratory illness. Patients should be encouraged to volunteer recent travel histories, and HCWs should elicit information regarding travel to affected areas. Public health and laboratory partners should be notified of suspect cases, as appropriate, during the diagnostic work-up, so that emerging pathogen screening, risk assessment, and advice can be guided in a timely manner.
